# Transcriptome profiling of drought responsive noncoding RNAs and their target genes in rice

**DOI:** 10.1186/s12864-016-2997-3

**Published:** 2016-08-08

**Authors:** Pil Joong Chung, Harin Jung, Dong-Hoon Jeong, Sun-Hwa Ha, Yang Do Choi, Ju-Kon Kim

**Affiliations:** 1 Graduate School of International Agricultural Technology and Crop Biotechnology Institute/GreenBio Science & Technology, Seoul National University, Pyeongchang, 25354 Korea; 2 Department of Agricultural Biotechnology, Seoul National University, Seoul, 08826 Korea; 3 Department of Life Science, Hallym University, Chuncheon, 24252 Korea; 4 Department of Genetic Engineering and Graduate School of Biotechnology, Kyung Hee University, Yongin, 17104 Korea

**Keywords:** *Oryza sativa*, Drought stress, lncRNAs, miRNA, Non-coding RNA, Putative target genes, RNA-seq

## Abstract

**Background:**

Plant transcriptome profiling has provided a tool for understanding the mechanisms by which plants respond to stress conditions. Analysis of genome-wide transcriptome will provides a useful dataset of drought responsive noncoding RNAs and their candidate target genes that may be involved in drought stress responses.

**Results:**

Here RNA-seq analyses of leaves from drought stressed rice plants was performed, producing differential expression profiles of noncoding RNAs. We found that the transcript levels of 66 miRNAs changed significantly in response to drought conditions and that they were negatively correlated with putative target genes during the treatments. The negative correlations were further validated by qRT-PCR using total RNAs from both drought-treated leaves and various tissues at different developmental stages. The drought responsive miRNA/target pairs were confirmed by the presence of decay intermediates generated by miRNA-guided cleavages in Parallel Analysis of RNA Ends (PARE) libraries. We observed that the precursor miR171f produced two different mature miRNAs, miR171f-5p and miR171f-3p with 4 candidate target genes, the former of which was responsive to drought conditions. We found that the expression levels of the miR171f precursor negatively correlated with those of one candidate target gene, but not with the others, suggesting that miR171f-5p was drought-responsive, with Os03g0828701-00 being a likely target. Pre-miRNA expression profiling indicated that miR171f is involved in the progression of rice root development and growth, as well as the response to drought stress. Ninety-eight lncRNAs were also identified, together with their corresponding antisense transcripts, some of which were responsive to drought conditions.

**Conclusions:**

We identified rice noncoding RNAs (66 miRNAs and 98 lncRNAs), whose expression was highly regulated by drought stress conditions, and whose transcript levels negatively correlated with putative target genes.

**Electronic supplementary material:**

The online version of this article (doi:10.1186/s12864-016-2997-3) contains supplementary material, which is available to authorized users.

## Background

There is growing concern regarding current and future environmental changes worldwide, such as increases in average air and sea temperatures and altered rainfall patterns, and the abiotic stresses that they impose on biological systems [1, 2]. Plant adaptations to such stresses involve complex signal transduction pathways [3], and elucidating the associated gene expression networks [4], in order to develop strategies to enhance the stress tolerance of crops [5–7], is an important objective of agricultural biotechnology.

Many studies have investigated plant stress tolerance using transcriptional profiling, thereby revealing differences between control and stress-treated plants in the relative expression levels of genes encoding stress response regulators and their target proteins [4, 8]. However, while typically more than 90 % of a eukaryotic genome is transcribed, only 1–2 % is translated into proteins [9], and indeed, in addition to stress-inducible regulatory proteins and transcription factors, microRNAs (miRNAs) are also known to regulate plant stress responses [10–12]. miRNAs are a class of small noncoding RNAs that regulate gene expression at the post-transcriptional level by mRNA cleavage or translational inhibition of the target gene [13]. There are currently >10,000 plant miRNA sequences from >120 plant species in the miRBase database (www.mirbase.org) [14] including 713 from rice (*Oryza sativa*) miRNA sequences. Several miRNAs have been reported to regulate drought-responsive genes [10, 15, 16], and it has been shown that rice miR159, miR169, miR395 and miR474 are drought-inducible, while the expression of miR156, miR168, miR170, miR172, miR396, miR397 and miR408 is suppressed by drought [13, 16]. In addition, miR171 and miR319 expression is either increased or repressed, depending on the specific drought conditions [15]. Drought-induced miRNAs downregulate their target transcripts, whereas drought-induced suppression of miRNAs results in the increased accumulation of their target transcripts [17, 18]. For example, miR169 is down-regulated under drought stress in *Arabidopsis thaliana*, whereas its target gene, *NFYA5,* is drought-induced [19]. Phenotypic analysis of mutants, or transgenic plants in which the expression of either stress-responsive miRNAs or their target genes have been manipulated, has been used to determine the role of miRNAs under different stress conditions [20, 21].

Another class of noncoding RNAs are the long noncoding RNAs (lncRNAs), which can be classified into five categories: i) sense and ii) antisense, when there is overlap of different transcripts in the same, or opposite, strand, respectively; iii) bidirectional, when the expression of an lncRNAs and a neighboring coding transcript on the opposite strand is initiated in close genomic proximity; iv) intronic, when it is derived wholly from within an intron of a second transcript; and v) intergenic, when it lies within the genomic interval between two genes [22]. Numerous lncRNAs have been associated with responses to abiotic stress, such as the expression of 1,832 lncRNAs that were reported to be regulated by various abiotic stresses in *A. thaliana* [23], 125 lncRNAs that were identified under drought and heat stress conditions in wheat (*Triticum aestivum*) [24] and several drought-responsive and tissue-specific maize (*Zea mays*) lncRNAs [25].

In this current study, RNA-sequencing (RNA-seq) transcript profiling was used to evaluate the levels of noncoding RNAs, including pri-miRNAs and lncRNAs, in well-watered control and drought-treated rice plants. A total of 66 drought-responsive miRNA precursors (24 drought-inducible and 42 drought-repressible), which have not previously been characterized in rice, were identified. The expression levels of some of these were shown, by qRT-PCR, to have a negative correlation with the expression of their candidate target genes. In addition, Parallel Analysis of RNA Ends (PARE) libraries from various rice tissues enabled the identification of decay intermediates generated by miRNA-guided cleavages [26], and a total of 98 drought-responsive lncRNAs and their sense or antisense transcripts were detected. The combined data sets suggest potential roles for specific rice miRNAs under drought conditions.

## Results

### Exposure of rice plants to conditions that mimic natural drought stress

**Fig. 1:**
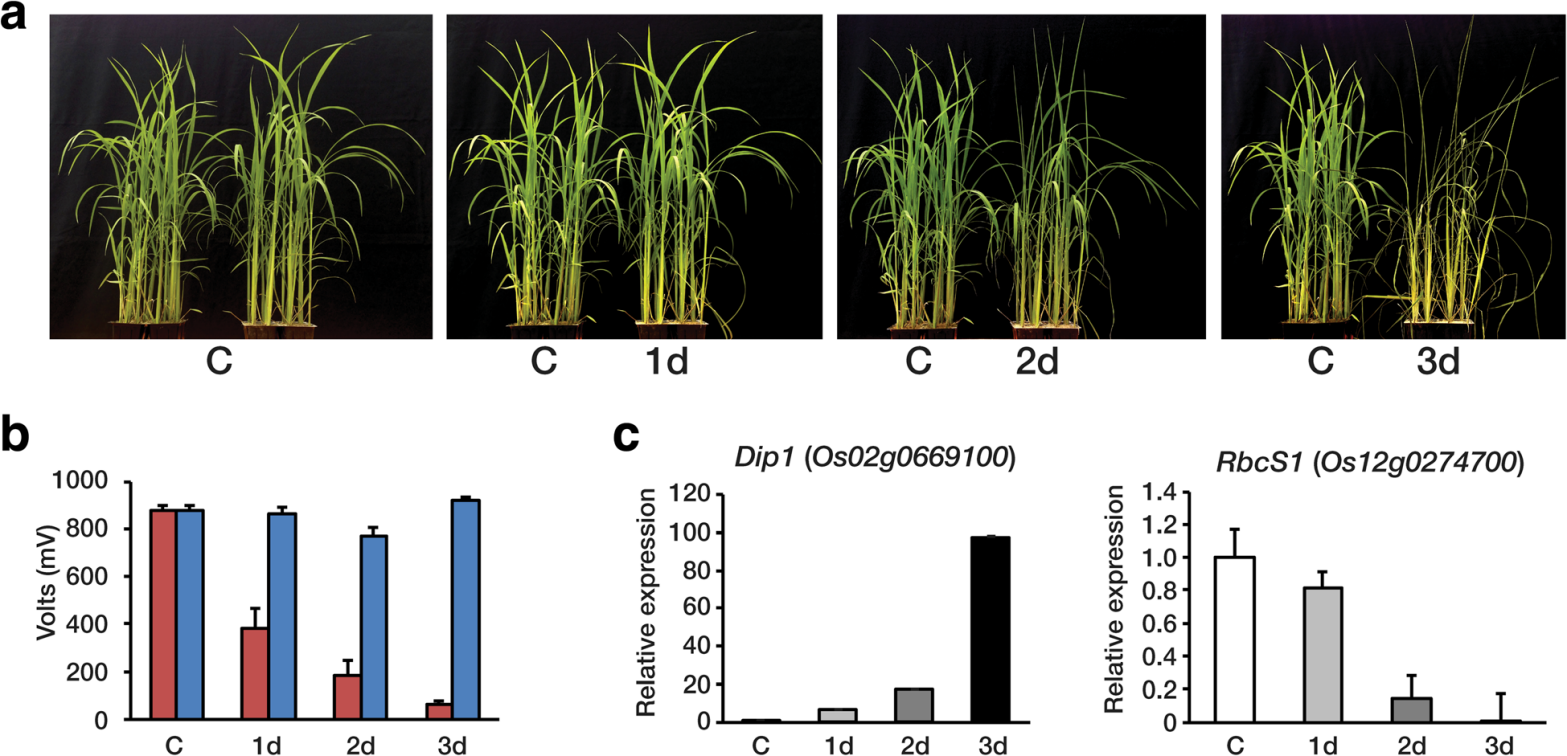
Drought response phenotype of rice in the vegetative state. **a** The phenotypic effect of progressive drought on wild type rice (*Oryza sativa* cv. Ilmi) at the vegetative growth stage. Drought stress was initiated 40 days after germination, and the plants shown are a well-watered control and at day 1, 2 and 3 after drought initiation. **b** Decrease in soil water content during drought treatment. Soil moisture in the pots was monitored using a SM150 Soil Moisture Sensor (Delta-T Devices Ltd). Volts (mV) is the SM150 output value. Blue bar, control; Red bar, drought condition. The conversion from SM150 reading (volts) to soil moisture (%) can be calculated by −0.0714 + 1.7190 V-3.7213 V^2^ + 5.8402 V^3^-4.3521 V^4^ + 1.2752 V^5^ (Delta-T Devices Ltd). **c** The transcript levels of *Dip1* and *RbcS1* in the leaves of drought-treated and well-watered control plants over a time course of exposure to drought were measured by qRT-PCR analysis. Values shown are the means ± SD of three independent experiments and are presented relative to the results from the control. *Dip1* (Dehydration Stress-Inducible Protein1, Os02g0669100) and *RbcS1* (Small subunit of Rubisco, Os12g0274700) served as stress marker genes

Rice plants were grown in a greenhouse for 5 weeks before being subjected to drought stress. To mimic natural drought stress, drought conditions were imposed by withholding water for 3 d, until a soil water content of < 10 % was measured. Leaves of drought stressed plants were compared to those of control plants grown under normal irrigation conditions. After a day, the soil moisture content dropped to 50 % of the initial soil capacity and rice plants started to show visual symptoms of drought-induced damage, such as leaf rolling (Fig. 1a and Fig. 1b). All the leaves from drought-treated plants showed a greater degree of leaf rolling as the level of drought stress increased. Consequently, the rice plants were severely affected by drought after 3 d (Fig. 1a). In addition to the phenotypic assessment, we measured the expression of the *Dip1* (Dehydration stress-inducible protein 1; Os02g0669100) and *RbcS1* (Small subunit of rubisco; Os12g0274700) genes, whose expression has been reported to be drought-inducible and drought-repressed, respectively [27]. *Dip1* expression was observed to increase at 1 d, and continued to increase up to 3 d, whereas transcript levels of *RbcS1* progressively decreased until 3 d after the imposition of drought conditions (Fig. 1c).

### RNA-seq analysis

Total RNA was extracted from the leaves of drought treated and well-watered plants and used to construct four RNA-seq libraries: one library from a well-watered control (C) and three libraries from drought-treated leaves (1 to 3 d). These were sequenced using an Illumina Hi-seq 2500 to identify differences in expression profiles among the different libraries. Sequence read information is summarized in Additional file 1: Table S1. Approximately 492 million single-end sequence reads were obtained and after quality trimming a total of 254 million sequence reads remained, corresponding to 24,667,603,889 bp (49 %). A flow chart of the sequencing process is shown in Additional file 2: Figure S1. A total of 81 % of the reads could be mapped to predicted gene regions. Raw sequence reads were trimmed to remove adaptor sequences and those with a quality lower than Q20 were also removed using the clc mapping tool (clc_ref_assemble 6 in the CLC ASSEMBLY CELL package).

Drought responsive genes were defined those that were differentially expressed between well-watered and drought-treated leaves, and we observed that among these genes, approximately twice as many were down-regulated by drought as were up-regulated in the 2 d and 3 d samples (Additional file 3: Figure S2). Far fewer genes were up-regulated in the 1 d sample. Of the 44,553 genes that could be annotated using the RAP-DB database (http://rapdb.dna.affrc.go.jp), 1,963 and 2,286 were up- and down-regulated, respectively, by more than 2-fold upon drought treatment for 1 d. Similarly, 8,070 and 12,518 genes were up- and down-regulated, respectively, after 2 d, and 7,888 and 17,746 genes, respectively, after 3 d. Of the genes identified as differentially expressed between treatments, 853 and 479 were up- and down-regulated, respectively, in all three drought treated samples (Additional file 4: Tabular data 1).

The assembled contigs were annotated using the gene ontology (GO) database BLAST mapping function (BLAST2GO) at the EMBL-EBI website (http://www.ebi.ac.uk/QuickGO/GAnnotation). Additional file 5: Figure S3 shows the genes that could be assigned at least one GO term in the three main GO categories, ‘biological process’, ‘cellular component’ and ‘molecular function’.

### Drought-responsive miRNAs and their candidate target genes

**Fig. 2:**
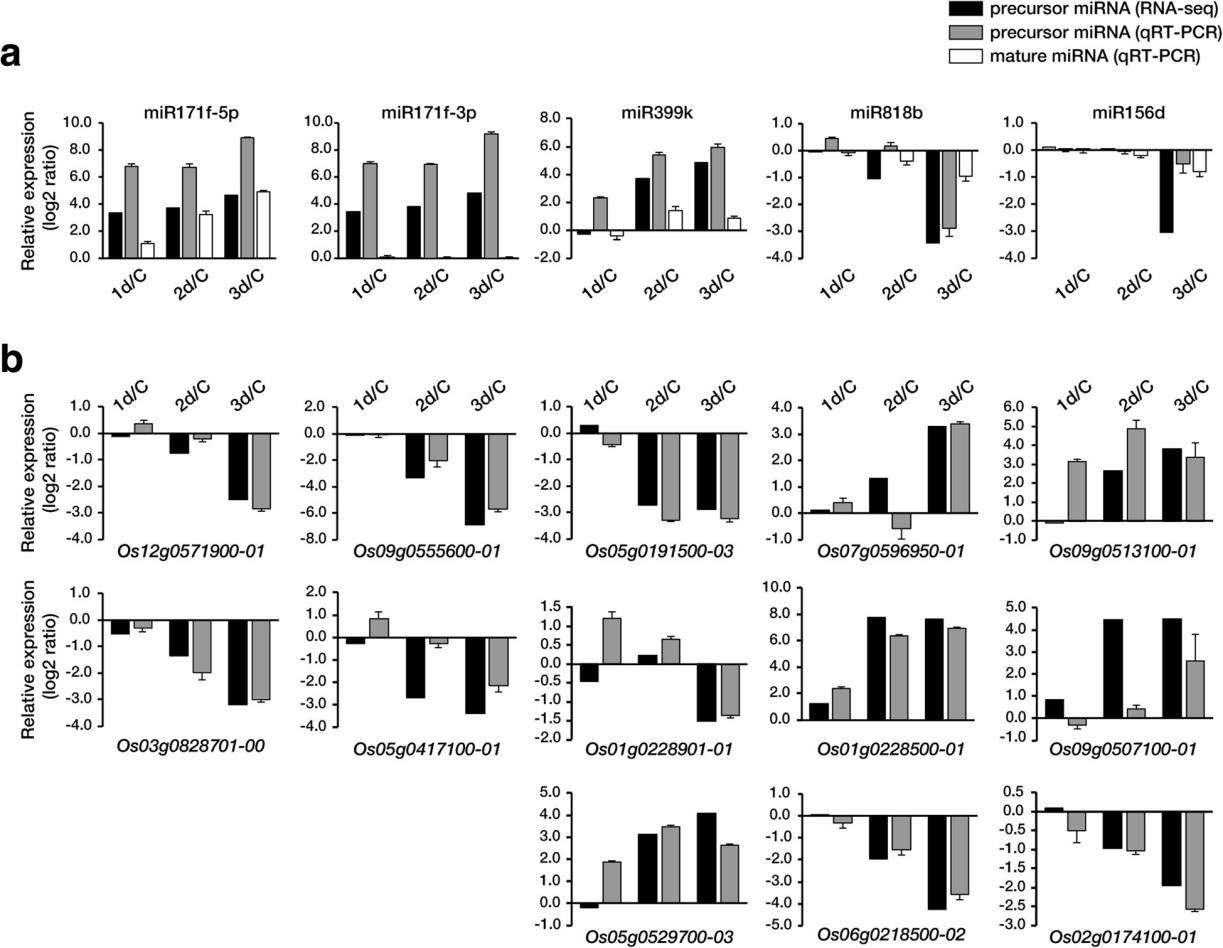
qRT-PCR confirmation of RNA-seq results examining gene expression in leaves from plants grown under well-watered and drought conditions. Changes in expression of precursor miRNAs, mature miRNAs (**a**) and putative target genes (**b**) as determined by qRT-PCR and compared with the RNA-seq data. The target genes of the miRNAs were predicted using the web tool, psRNATarget (http://plantgrn.noble.org/psRNATarget/). Bar indicated as mean values ± SD (standard deviation) of three independent experiments

**Fig. 3:**
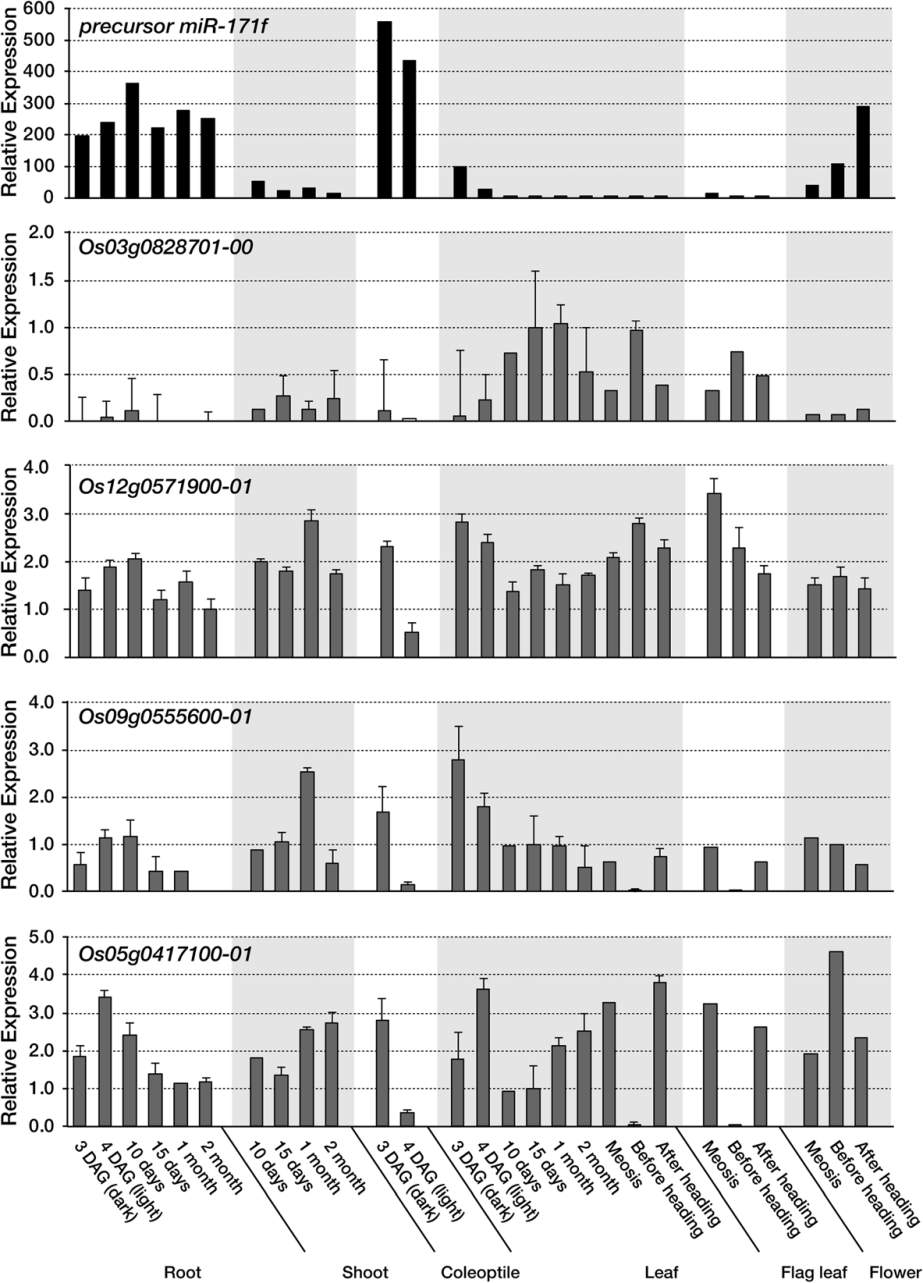
Expression analysis of the drought-responsive miRNA precursor miR-171f and its putative target genes (Os03g0828701-00, Os12g0571900-01, Os09g0555600-01 and Os05g0417100-01) in various plant tissues at different developmental stages. Rice seeds were germinated and grown on MS (Murashige and Skoog) medium in the dark for 3 d (3 DAG, day after germination) and then in the light for 1 d at 28 °C (4 DAG). Seedlings were then transplanted into soil pots, and grown in the greenhouse for 10 d, 15 d, 1 month and 2 month until meiosis (meiosis), just prior to heading (before heading, BH) and right after heading (after heading, AH). qRT-PCR analyses of each gene were performed with the indicated tissues at the different developmental stages. Rice *Ubi1* (AK121590) was used as an internal control. C, coleoptiles; R, roots; L, leaves; FL, flag leaves; F, flowers (panicles). Bar indicated as mean values ± SD (standard deviation) of three independent experiments

To date, 592 rice miRNA precursors (pre-miRNAs) encoding 713 mature miRNAs have been reported (www.mirbase.org), and in this current study we identified a total of 113 pre-miRNAs in the RNA-seq data sets. Of those, 26 pre-miRNAs were constitutively expressed under both normal and drought conditions at high levels, whereas 21 pre-miRNAs were expressed at low levels (Additional file 6: Table S3). In addition, the expression levels of 24 pre-miRNAs increased considerably upon exposure to drought stress conditions, while those of 42 were substantially decreased (Additional file 7: Table S2). These drought-responsive miRNAs and their putative target genes, predicted by the web tool psRNATarget (http://plantgrn.noble.org/psRNATarget/), are listed in Additional file 8: Tabular data 2. For 18 of the pre-miRNAs that were highly up-regulated in response to drought stress, their putative target genes showed a concomitant decrease in transcript levels. Conversely, for 20 precursor miRNAs that were strongly down-regulated by the drought treatment, a concomitant increase in transcript levels of their putative target genes was observed (in Additional file 8: Tabular data 2). To validate the RNA-seq results and the inverse correlations in expression levels between the miRNAs and their target genes, qRT-PCR was carried out using total RNAs from control and drought-treated leaves. Expression levels of the miRNAs and their candidate target genes were again seen to be inversely correlated (Fig. 2), consistent with their expected function in cleaving the target mRNAs. qRT-PCR was also used to determine the correlation in expression of the precursor and mature miRNAs, and we observed that the expression patterns of the drought-responsive miR171f-5p, miR399k, miR818b and miR156d precursors correlated well with those of the mature miRNAs (Fig. 2a). Interestingly, while pre-miR171f and miR171f-5p showed a drought-inducible expression pattern, the expression of miR171f-3p, which is another mature miRNA derived from pre-miR171f, was affected by drought. However, this could be due to the fact that the miR171f-3p sequence is also encoded by other members of the miR171 family, such as pre-miR171b, pre-miR171c, pre-miR171e and pre-miR171f, which are also not responsive to drought. It is also possible that processing of pre-miR171f to generate miR171-5p or miR171-3p is differentially regulated by drought. Since miR-171f-5p and miR-171f-3p have different sets of target genes, we measured the expression levels of the miR171f precursor and the putative target transcripts (Os03g0828701-00 and Os12g0571900-01 for miR-171f-5p; Os09g0555600-01 and Os05g0417100-01 for miR-171f-3p) in various rice tissues at different developmental stages by qRT-PCR (Fig. 3) [28]. The precursor miR-171f accumulated at high levels in roots, coleoptiles and flowers. Conversely, transcripts of Os03g0828701-00, a target of miR-171f-5p, were observed in leaves, but not in roots and flowers. Thus, the expression pattern of the miR-171f precursor has an inverse correlation with that of its corresponding target gene, Os03g0828701-00, but not with the other predicted target genes, Os12g0571900-01, Os09g0555600-01 and Os05g0417100-01. Considering the results presented in Figs. 2 and 3, we concluded that miR-171f-5p is drought-responsive, with Os03g0828701-00 being a likely target gene.

Parallel Analysis of RNA Ends (PARE), also known as RNA degradome analysis, enables high-throughput miRNA target identification. To validate predicted targets of the drought-responsive miRNAs, rice PARE data were obtained from the NCBI Gene Expression Omnibus with accession numbers GSM455938, GSM455939, GSM476257 and GSM434596 (http://www.ncbi.nlm.nih.gov/geo/) [29]. PARE sequences matching to cleavage products, starting between base 10 and 11 from the 5′ end of the predicted miRNA pairing, were considered to be evidence of miRNA-guided cleavage (Table 1). In total, 32 target cleavages guided by 21 drought-responsive miRNAs were identified.

**Table 1:** Drought responsive precursor miRNAs with their candidate target genes and their expression patterns

Gene ID	^a^RPKM				^b^Log2 Ratio			^c^Mature miRNA/target sequence	^d^PARE sequence	PARE libraries			
	C	d1	d2	d3	1d/C	2d/C	3d/C			^e^A	^f^B	^g^C	^h^D
Inducible miRNAs/targets													
miR-399k	119,291	99,410	1,530,906	3,519,097	−0.26	3.68	4.88	3′-GCCCCGUUUAAAGGAAACCGU-5′					
Os05g0557700-01	25,889	23,611	8,802	9,104	−0.13	−1.56	−1.51	737-CUGGGCAAAUCUCCUUUGGCA-757	TCCTTTGGCAAAATACCTAT	0	0	1	1
miR-415	234,165	109,765	160,988	1,529,388	−1.09	−0.54	2.71	3′-GACGAGACGAAGACAAGACAA-5′					
Os04g0550200-01	9,337	2,36	1,749	273	−1.98	−2.42	−5.10	789-UUACUCUGCUUCUGCUCUGUU-809	CTGCTCTGTTCTCTTTCTTC	0	0	0	1
Os10g0500500-01	13,806	11,519	7,428	3,613	−0.26	−0.89	−1.93	976-UGCUCUGCAUUUCUUCUGUU-995	TTCTTCTGTTACTCATTCGA	0	2	0	0
miR-168a	329,822	678,870	1,513,995	981,069	0.11	1.27	0.64	3′-AGGGCUAGACGUGGUUCGCU-5′					
Os02g0831600-01	5,715	5,283	1,584	687	−0.11	−1.85	−3.06	446-UCCCGAGCUGCGCCAAGCAA-465	CGCCAAGCAATAATGGAAGC	0	0	2	7
Os03g0687000-02	11,281	10,412	3,293	1,896	−0.12	−1.78	−2.57	1807-UCAUGAUCUGCGCCAAGUGG-1826	CGCCAAGTGGTACAGGTTCA	0	0	0	1
Os03g0687000-01	9,006	8,713	2,571	1,591	−0.05	−1.81	−2.50	1905-UCAUGAUCUGCGCCAAGUGG-1924	CGCCAAGTGGTACAGGTTCA	0	0	0	1
miR-821c	27,730	47,537	376,336	364,452	0.78	3.76	3.72	3′-AGUUGAAAAAACAACUACUGAA-5′					
Os10g0412600-01	14,944	16,110	4,884	334	0.11	−1.61	−5.48	685-UGAACUUUUUUAUUGGUGAUUC-706	TTGGTGATTCCCTCTAATGT	0	0	0	1
miR-171f-3p	8,297	82,972	107,863	207,429	3.32	3.70	4.64	3′-CUAUAACCGUGCCGAGUUAGU-5′					
Os09g0555600-01	2,031	1,926	380	60	−0.08	−2.42	−5.07	1488-GGUAUUGGCAUUGCUCAAUUA-1508	TGCTCAATTATGGGCTAAAG	0	0	0	1
miR-816	150,534	95,795	301,069	136,849	−0.65	1.00	−0.14	3′-CAACAUCAUUUUAUACAGUG-5′					
Os01g0338100-00	1,722	1,227	701	534	−0.49	−1.30	−1.69	277-AUUGUUGUAGAAUAUGUCAC-296	AATATGTCACTGACCTGGTC	0	0	0	1
miR-166c-5p	16,860	50,580	25,290	42,150	1.58	0.58	1.32	3′-GAGCCUGGUCUGUUGUAAGG-5′					
Os03g0823100-01	41,418	36,220	21,357	6,526	−0.19	−0.96	−2.67	784-CUUGGACCAGCCAAUAUUUU-803	CCAATATTTTCCTTCTATTT	0	0	1	1
miR-166c-3p	16,860	50,580	25,290	42,150	1.58	0.58	1.32	3′-CCCUUACUUCGGACCAGGCU-5′					
Os12g0612700-01	667	751	573	121	0.17	−0.22	−2.47	874-UGGGAUGAAGCCUGGUCCGG-893	CCTGGTCCGGATTCCATTGG	0	0	53	73
miR-167g	-	25,701	89,953	12,850	-	-	-	3′-GUCUAGUACGACCGUCGAAGU-5′					
miR-167b	6,465		19,394	12,929	- -	1.58	1.00	3′-UCUAGUACGACCGUCGAAGU-5′					
Os06g0129100-01	21,789	14,688	2,477	275	−0.57	−3.14	−6.31	1085-UGUUCAUGCCGGCAGCUUCA-1104	GGCAGCTTCAGGCTCCAGGT	0	0	0	10
Os07g0481400-01	10,354	8,139	4,789	1,387	−0.35	−1.11	−2.90	2770-UAGAUCAUGCUGACAGCCUCA-2790	GACAGCCTCAAAACAATTGA	0	0	1	6
miR-159b	11,210	67,260	67,260	-	2.58	2.58	-	3′-GUCUCGAGGGAAGUUAGGUUU-5′					
Os06g0605600-01	3,381	3,623	4,557	934	0.10	0.43	−1.86	403-UAGAGCUCCCUUCACUCCAAU-423	TCACTCCAATATCCCAACTA	0	3	0	16
Os03g0683866-00	5,268	5,001	4,429	1,662	−0.07	−0.25	−1.66	1320-UAAAGCUGCCUUCAGUCCAGA-1340	TCAGTCCAGAATATGGGCTT	0	0	0	1
miR-159f	5,605	11,210	28,025	-	1.00	2.32	- -	3′-AUCUCGAGGGAAGUUAGGUUC-5′					
Os06g0605600-01	3,381	3,623	4,557	934	0.10	0.43	−1.86	403-UAGAGCUCCCUUCACUCCAAU-423	TCACTCCAATATCCCAACTA	0	3	0	16
miR-169f	-	6,198	43,389	198,351	- -	- -	- -	3′-AUCCGUUCAGUAGGAACCGAU-5′					
Os02g0776400-01	1,807	1,507	469,	60	−0.26	−1.94	−4.91	983-UAGGCAAUUCAUCCUUGGCUU-1003	TCCTTGGCTTAAGTTTCATG	6	5	6	66
Os03g0411100-01	12,413	14,677	4,257	2,061	0.24	−1.54	−2.59	1242-UGGCAAUUCAUCCUUGGCUU-1261	TCCTTGGCTTATGAAGTATC	28	42	37	156
miR-156i	-	-	23,416	23,416	- -	- -	- -	3′-CACGAGUGAGAGAAGACAGU-5′					
Os06g0663500-00	3,829	3,070	3,323	867	−0.32	−0.20	−2.14	749-GUGCUCUCUCUCUUCUGUCA-768	TCTTCTGTCAGCTAGTTCAA	0	5	1	35
Os02g0174100-01	2,114	2,279	1,071	564	0.11	−0.98	−1.95	2221-GUGCUCUCUCUCUUCUGUCA-2240	TCTTCTGTCATCTAGTTCTT	0	2	0	11
Os02g0139400-01	13,403	16,632	13,100	1,392	0.31	−0.03	−3.27	1869-AUGCUCUCUCUCUUCUGUCA-1888	TCTTCTGTCAATCGATTCAG	0	5	25	34
Repressible miRNAs / targets													
miR-530	1,433,087	241,496	231,823	84,299	−1.77	−2.63	−4.09	3′-AUCCACGUCCACGUUUACGU-5′					
Os04g0603200-01	28,344	24,091	55,324	69,156	−0.23	0.96	1.29	653-CAGAUGAAGGUGCAAAUGCA-672	TGCAAATGCAGGAGCTGTAA	0	0	0	1
Os03g0296700-02	334	434	534	568	0.38	0.68	0.77	440-UGGAUGCUGGUGCAGAUGCA-459	TGCAGATGCACCGTTCTGAT	0	0	1	0
miR-399e	169,670	107,160	17,860	8,930	−0.66	−3.25	−4.25	3′-CCCGUUUAGAGG-AAACCGU-5′					
Os04g0415000-01	5,599	8,625	25,400	18,132	0.62	2.18	1.70	491-GGGCAAUUCUCCGUUUGGCA-510	CCGTTTGGCAGAAGATCAAC	0	0	0	1
miR-156f	1,501,297	1,370,996	657,172	436,226	−0.13	−1.19	−1.78	3′-CACGAGUGAGAGAAGACAGU-5′					
miR-156d	1,070,078	1,159,932	1,110,921	130,697	0.12	0.05	−3.03	3′-CACGAGUGAGAGAAGACAGU-5′					
miR-156g	136,247	364,020	657,790	19,159	0.09	−0.45	−1.91	3′-CACGAGUGAGAGAAGACAGU-5′					
miR-156j	351,247	364,020	657,790	19,159	0.05	0.91	−4.20	3′-CACGAGUGAGAGAAGACAGU-5′					
Os01g0922600-01	1,610	2,024	20,929	4,295	0.33	3.70	1.42	620-GUGCUCUCUCUCUUCUGUCA-639	TCTTCTGTCAGACAACCCCA	0	0	2	19
Os09g0507100-00	42	75	941	950	0.83	4.47	4.49	1034-GUGCUCUCUCUCUUCUGUCA-1053	TCTTCTGTCATCCCCGGCCA	0	0	2	1
miR-815a	25,391	-	12,696	12,696	-	−1.00	−1.00	3′-GGUUAGAGGAGUUAGGGGAA-5′					
miR-815c	511,525	531,986	225,071	71,613	0.06	−1.18	−2.84	3′-GGUUAGAGGAGUUAGGGGAA-5′					
Os08g0465800-01	9,970	9,526	61,668	74,744	−0.07	2.63	2.91	1896-CCAAUCUCCUUCCUCCUCUU-1915	TCCTCCTCTTTTTAATCTCT	0	0	0	1
miR-159a	395,153	360,286	96,851	65,859	−0.13	−2.03	−2.58	3′-UCUCGAGGGAAGUUAGGUUU-5′					
Os03g0331700-02	7,076	3,394	22,641	50,780	−1.06	1.68	2.84	1513-UGAGUUCCCUUCAUUCCAAA-1532	TCATTCCAAAAGCTTAATTG	0	0	0	1
miR-393b	151,675	175,623	127,726	31,932	0.21	−0.25	−2.25	3′-UAGUUACGCUAGGGAAACCU-5′					
Os05g0150500-00	5,537	4,773	20,300	36,796	−0.21	1.87	2.27	1556-GACAAUGCGAUCCCUUUGGA-1575	TCCCTTTGGATGTCGTCGTG	16	18	88	655
miR-528	227,512	287,384	23,949	-	0.34	−3.25	-	3′-GAGGAGACGUACGGGGAAGGU-5′					
Os07g0570550-00	290	522	1,334	1,566	0.85	2.20	2.43	180-CUCCUCUGC-UGCCCCUUCCA-199	GCCCCTTCCATGGCGCCCGC	0	0	205	0
miR-166d	92,729	33,720	84,299	16,860	−1.46	−0.14	−2.46	3′-CCCUUACUUCGGACCAGGCU-5′					
Os03g0640800-01	1,189	1,224	4,376	8,977	0.04	1.88	2.92	956-UGGGAUGAAGCCUGGUCCGG-975	CCTGGTCCGGATTCCATTGG	0	0	53	73
Os10g0480200-02	1,638	1,282	2,956	3,158	−0.35	0.85	0.95	922-UGGGAUGAAGCCUGGUCCGG-941	CCTGGTCCGGATTCGTTTGG	0	3	12	179

^a^RPKM, Reads Per Kilobase of transcript per Million mapped reads; ^b^log2 ratio, log_2_(drought treatment / control); ^c^bases underlined indicated potential cleavage sites; ^d^PARE sequence matching to cleavage products, starting between base 10 and 11 from the 5′ end of the predicted miRNA pairing; ^e^A (SC938), PARE library of rice wild type seedling degradome, GSM455938 (GEO Accession number); ^f^B (INF939), PARE library of rice wildtype inflorescence degradome, GSM455939; ^g^C (INF9311a), PARE library of rice inflorescence (93-11) wildtype degradome, GSM476257; ^h^D (NPBs), PARE library of rice 3-week-old seedlings wildtype degradome, GSM434596 [29]

### Drought-responsive lncRNAs and their Natural Antisense Transcripts (NATs)

In this study, 98 drought-responsive lncRNAs (31 up- and 67 down-regulated, respectively, with a log_2_ ratio ≥ 2.0 and ≤ −2.0) with over 1 kb in length and their cognate antisense transcripts were identified (Additional file 9: Table S4 and Additional file 10: Tabular data 3). A subset of the lncRNAs comprise the class ‘Natural Antisense Transcripts’ (NATs), which are complementary to other endogenous transcripts of coding or noncoding genes. These can be transcribed in *cis* from the same genomic locus as the target mRNA, or in *trans* from a separate locus. Of the 98 lncRNAs, 58 pairs were determined to be *cis*-NATs, i.e., two or more genes within the NAT pair that are located on the opposite strands of the same genomic locus. Additional 6 regions of bidirectional transcription were also found, which were arranged in a convergent orientation to the 5′ end or with the 3′ end overlapping. Additionally, 22 intergenic and 5 sense lncRNAs (Additional file 9: Table S4) were identified, and we determined that the expressions of most of the lncRNAs was consistent with expression of the nearby coding or noncoding transcripts, while the expression of two NATs and their candidate target genes were inversely correlated: the NAT Os02g0250700-01 and its candidate target gene Os02g0250600-01 (late embryogenesis abundant protein, LEA), and the NAT Os02g0180800-01 and its target gene Os02g0180700-01 (cinnamoyl-CoA reductase). The former pair shows a head-to-head genomic configuration while the latter shows a tail-to-tail configuration.

## Discussion

Compared with natural drought conditions, where dehydration is typically gradual and progressive, experimental treatments to induce drought are often relatively severe and/or rapid. For example, widely used methods involve air drying with excised leaf disc or treating them with polyethylene glycol [30, 31]. Such treatments are liable to cause osmotic stress rather than drought stress, and indeed it can be difficult to distinguish between these types of stress. In this current study, we sought to analyze the molecular response of rice plants subjected to a mild drought stress, thereby mimicking natural drought conditions. Transcriptome profiling was performed of leaves from rice plants grown at either 75 %, 40 %, 10 % or 7 % residual soil moisture content (Fig. 1), where drought stress damage was carefully monitored using the expression of *Dip1* and *RbcS1* as markers for drought-inducible and drought-sensitive expression, respectively. We then examined the RNA-seq data to identify differentially expressed genes involved in drought responses.

Amongst the genes that were found to be associated with the drought response and that were differentially expressed between well-watered and water-deficit conditions, we identified both drought induced genes, including late embryogenesis abundant (Os06g0324400-01, Os03g0322900-00, Os06g0110200-01), calcium-dependent membrane targeting domain protein (Os04g0476600-01), and drought repressed genes, such as A-type response regulator, (Os11g0143300-01, Os12g0139400-01) (Additional file 4: Tabular data 1). In addition, a total of 66 drought-responsive pre-miRNAs were identified, 24 which were drought-induced and 42 of which were drought-repressed by more than 2-fold. Of the 66 rice pre-miRNAs, 41 are identified as being drought-responsive for the first time in this study (Table 1). Sixty-six pre-miRNAs could be assigned to 29 miRNA families, while 10 did not belong to any family. Interestingly, two members of the miR399 family, pre-miR399k and pre-miR399d, were up-regulated by drought stress, while other two members, pre-miR399e and pre-miR399i, were down-regulated by drought stress. Similarly, some members of the miR156, miR159, miR167 and miR169 families (pre-miR156b/i, pre-miR159b/f, pre-miR166a/b/c, pre-miR167b/g, pre-miR169f/p) were up-regulated while others (pre-miR156d/f/g/j, pre-miR159a, pre-miR166d, pre-miR167d/e, pre-miR169a/b/h/l/m/q) were down-regulated by drought stress. These results suggest that members of the same miRNA family are functionally diverse during drought responses. A number of drought-responsive miRNAs have been identified [32–34]; however, we found that the expression patterns of 14 pre-miRNAs (miR156i/b/f, miR168a, miR172a/d and miR169a/b/h/l/m/q, miR171e, miR393b) were different under the drought treatments used in this study from those previously reported [10, 15]. This discrepancy may be due to different ages of the tissues used or the way in which the drought treatments were imposed. It is also possible that the expression patterns between the mature miRNAs and pre-miRNAs are different during drought conditions.

Experimental validation of the putative miRNA paired target genes has been a major focus in the investigation of miRNA function [35]. Of the differentially expressed precursor miRNAs and their putative target genes, 5 were confirmed by qRT-PCR (Fig. 2), and the expression level of these genes confirmed the accuracy of the RNA-seq data. Under drought-stress conditions, the transcription of stress-responsive miRNAs and their putative targets can be independently regulated. Indeed, the expression patterns of some of the pre-miRNAs were positively correlated with those of their target genes (Additional file 8: Tabular data 2). Conversely, many drought-responsive miRNAs and their targets showed a negative correlation in their expression patterns, i.e., drought-induced miRNAs downregulated their target mRNAs, while drought-repressed miRNAs upregulated their target mRNAs (Fig. 2 and Additional file 8: Tabular data 2). Thus, a given target gene may either promote or suppress processes during stress adaptation responses. Drought-responsive miRNA-mediated target cleavages were also confirmed by analyzing publically available PARE data (Table 1). Some miRNAs, such as miR819d, miR171f, miR156, miR530 and miR819i, have a large number of putative target genes. Additional file 8: Tabular data 2 shows that the candidate target genes were up- or down-regulated in an opposite manner to the change in miRNA expression, e.g., miR171f and its targets, Scarecrow-like 6 and the MORN motif containing protein, and miR156 and its target squamosa-promoter binding protein. miR171f has previously been observed to be responsive to salt, drought and cold stress in *A. thaliana* [36], and two mature miR171fs, miR171f-3p and miR171f-5p, were reported to be down-regulated under drought conditions in rice [33]. However, our results showed that the pre-miR171f and the mature miR171f-5p, but not miR171f-3p, were drought-inducible and that the expression level of the candidate target gene, Os03g0828701-00, was inversely correlated with that of miR171f-5p under drought conditions. The negative-correlation patterns between miR171f and Os03g0828701-00 were also found at various developmental stages of rice (Fig. 3), thereby providing insights into the function of miR171f, especially miR171f-5p, in the adaptive response of plants to drought stress at various development stages. Pre-miRNA expression profiling with RNA-seq and qRT-PCR of various developmental stages reveals that miR171f is involved in rice root growth and development as well as in responses to drought. Recently, it has been shown that virus infection specifically induces miR17f-5p expression in rice [32]. Given that mature the miR171f-3p sequence is also encoded by several other members of the miR171 family and that it is conserved to recognize target genes encoding GRAS family transcription factors, the pre-miR171f / Os03g0828701-00 module may be specifically developed to be involved in drought responses, developmental processes, and resistance to viral infection.

It was recently reported that a total of 37,238 long noncoding natural antisense transcripts (lncNATs) are associated with 70 % of the annotated mRNAs in *A. thaliana* [37]. In addition, 125 putative stress responsive lncRNAs from wheat have been reported [24], as well as 20,163 putative maize lncRNAs [38]. Zhang et al. identified 2,224 lncRNAs by sequencing strand-specific RNAs from various rice organs, including anthers, pistils, seeds, and shoots [39]. We found a total of 98 lncRNAs whose expression changed in response to drought (31 up-regulated and 67 down-regulated) and their expression levels were positively correlated with those of their putative target genes. Interestingly, two lncRNAs, Os02g0250700-01 and Os02g0180800-01, are bidirectional, and their potential targets are present on the neighboring opposite strand. Os02g0250700-01 and its target gene, Os02g0250600-01 (late embryogenesis abundant protein; LEA) was shown to share a single bidirectional promoter, and their expression is inversely correlated, as was the expression of Os02g0180800-01 and its target gene, Os02g0180700-01 (cinnamoyl-CoA reductase) under drought conditions.

Noncoding RNAs have been identified in many plant species, such as *A. thaliana*, maize, wheat, soybean (*Glycine max*) and rice; however, functional analysis is still challenging. Here, we identified drought responsive noncoding RNAs of the miRNA and lncRNA categories. The identification and expression pattern analysis of the rice precursor miRNAs and lncRNAs represents a resource for investigating how the extensive set of noncoding RNAs in the genome function and interact during drought stress and in regulating development.

## Conclusions

In this current study, we identified drought responsive noncoding RNAs by using RNA-seq profiling on well-watered control and drought-treated rice plants. A total of 66 drought-responsive miRNA precursors (24 up-regulated and 42 down-regulated), which have not previously been characterized in rice, were identified. The expression levels of some of these were shown to have a negative correlation with those of their candidate target genes. Those miRNA/target pairs were further validated by Parallel Analysis of RNA Ends (PARE) libraries from various rice tissues that enabled us to identify decay intermediates generated by miRNA-guided cleavages. In addition, a total of 98 drought-responsive lncRNAs (31 drought-inducible and 67 drought-repressible) and their sense or antisense transcripts were detected. The combined data sets suggest potential roles for specific rice noncoding RNAs under drought conditions.

## Methods

### Plant materials and drought-stress treatment

Rice (*Oryza sativa* cv. Ilmi) was germinated on MS (Murashige and Skoog) media at 28 °C for 4 days, and transplanted into soil pots (4 × 4 × 6 cm; 3 plants per pot) and grown in a greenhouse (37°32'51.3"N 128°26'26.6"E). Multiple pots of each rice were divided into 2 sets, one for drought and one for well-watered treatment conditions. Five weeks after transplanting to soil, total leaves of 10 whole plants growing in 4 pots were pooled and kept in liquid nitrogen for C, and then water was withheld from all the pots. The soil water content in each pot was adjusted to approximately 75 %. Soil moisture was monitored during the drought treatment using a Soil Moisture Sensor SM150 (Delta-T Devices, UK). After one, two and three days without watering, total leaves of 10 whole plants growing in 4 pots were pooled and kept in liquid nitrogen for 1 d, 2 d, and 3 d, respectively. Total RNA was extracted from pooled leaves of C, 1 d, 2 d, or 3 d. We grew an independent group of plants similar to above, and measured water content and expression levels of drought responsive marker genes using qRT-PCR as shown in Additional file 11: Figure S4. As a result, we prepared 2 independent sets of plants for drought treatments; one for RNA-seq (Fig. 1) and the other for qRT-PCR validation (Additional file 11: Figure S4).

### RNA extraction, RNA-seq library construction and sequencing

Total RNA was extracted from rice leaves using Trizol reagent (Invitrogen) and purified with an RNeasy Mini Kit (Qiagen). Contaminating genomic DNA was removed from the sample by treating with DNase I (Invitrogen), according to the manufacturer’s instructions. A modified TruSeq method was used to construct strand-specific RNA-seq libraries, with different index primers [40], and libraries were sequenced with an Illumina HiSeq 2500 system at the National Instrumentation Center of Environmental Management College of Agriculture and Life Science, Seoul National University (NICEM), as previously described [40]. Single-end sequences were generated and raw sequence reads were trimmed to remove adaptor sequences, and those with a quality lower than Q20 were removed using the clc quality trim software (CLCBIO). Duplicate paired short reads were removed using FastUniq [41], and all reads were assembled with the clc_ref_assemble 6 (version 4.06) program, using annotated gene and noncoding RNA sequences from the rapdb (http://rapdb.dna.affrc.go.jp) and ncRNA (http://www.ncrna.org) databases, respectively. The data set can be obtained from GEO database with series accession number GSE80811 for RNA-seq data.

### Quantitative RT-PCR validation of transcript abundance

One μg total RNA was reverse transcribed with oligo dT primers using 200 U of the RevertAid M-MuLV Reverse Transcriptase (Thermo Scientific, #K1621) for 60 min at 42 °C, and then the reaction was terminated by incubating for 5 min at 70 °C. Subsequent qRT-PCR was performed with first-strand cDNA as a template using gene-specific primer pairs and 2x Real-Time PCR smart mix (SolGent, SRH72-M10h) with EvaGreen (SolGent, 31000-B500). Reactions were performed at 95 °C for 15 min, followed by 40 cycles of 95 °C for 20 s, 60 °C for 20 s, and 72 °C for 30 s, in a 20 μl reaction mixture containing 1 μl of 20x EvaGreen, 10 μM primers, and ROX reference dye. Thermocycling and fluorescence detection were performed uisng a Strategene Mx300p real-time PCR machine and Mx3000p software version 2.02 (Stratagene). The *Ubi1* (AK121590) gene was used to verify equal RNA loading for the qRT-PCR analysis and as a reference in the RT-PCR. For detecting and quantifying mature miRNAs, stem-loop reverse transcription and RT-PCR of miRNAs was performed as described in Varkonyi-Gasic et al. and Chen et al. [42, 43]. Two hundred ng of total RNA was treated with RNAase-free DNase I (Promega), and transcribed into cDNA using gene specific RT primers and a thermostable reverse transcriptase (Invitrogen). First, the miRNA-specific stem-loop RT primer was hybridized to the miRNA and reverse transcribed. Reactions were performed at 16 °C for 45 min, followed by 60 cycles of 30 °C for 45 s, 42 °C for 45 s, and 50 °C for 1 s, in a 20 μl mixture containing 50 U Superscript III RT (Invitrogen), 4 U RNaseOUT (Invitrogen) and 1 μM stem-loop RT primer. Next, RT products were quantified using qRT-PCR with a miRNA specific forward and universal reverse primer. The rice *U6* small nuclear RNA (snRNA) gene was used to verify equal RNA loading for the qRT-PCR analysis and as a reference. A list of primers used in these experiments is available in Additional file 12: Table S5. All qRT-PCR results are representative from at least two biological repeats, each based on three technical repeats.

## Abbreviations

lncRNAs, long noncoding RNAs; miRNAs, micro RNAs; qRT-PCR, quantitative reverse transcribed polymerase chain reaction; RNA-seq, RNA sequencing

## Additional files

Additional file 1: Table S1. Information about the RNA-seq data obtained by Illumina Hi-seq 2500 sequencing. (XLSX 12 kb) Additional file 2: Figure S1. Flowchart of RNA-seq analysis. (TIF 9814 kb) Additional file 3: Figure S2. Heat Map of the differentially expressed coding (**a**) and noncoding genes (**b**) under drought conditions. (TIF 5474 kb) Additional file 4: Tabular data 1. Drought responsive genes and their expression patterns under well-watered and drought conditions. (XLSX 8528 kb) Additional file 5: Figure S3. Differentially expressed transcripts were classified into 3 main GO categories: Biological processes, Cellular components and Molecular functions. (TIF 8355 kb) Additional file 6: Table S3. List of miRNAs that are constitutive pattern of high expression and low expression level. ^1^ Bold in miRNAs ID, rice specific drought responsive miRNAs; ^2^ RPKM, Reads Per Kilobase of transcript per Million mapped reads; ^3^ C, control; ^4^ 1d, drought treatment for 1 day; ^5^ 2d, drought treatment for 2 days; ^6^ 3d, drought treatment for 3 days; ^7^ log2 ratio, log_2_(drought treatment / control), ^8^ Red, up-regulation by drought; Blue, down-regulation by drought Abbreviations: At, *Arabidopsis thaliana*; Bd, *Brachypodium distachyon*; Gm, *Glycine max*; Hv, *Hordeum vulgare*; Mt*, Medicago truncatula*; Me, *Manihot esculenta*; Pv, *Phaseolus vulgaris*; Peu, *Populus euphratica*; Ptc, *Populus trichocarpa*; Ppe, *Prunus persica*; Pte: *Populus tremula*; Pto, *Populus tomentosa*; Td, *Triticum dicoccoides*; Tt, *Triticum turgidum*; Os, *Oryza sativa*; Vu, *Vigna unguiculata*; Zm, *Zea mays* [10, 15]. (XLSX 24 kb) Additional file 7: Table S2. Drought responsive precursor miRNAs and their expression patterns under well-watered and drought conditions. ^1^ Bold in miRNAs ID, rice specific drought responsive miRNAs; ^2^ RPKM, Reads Per Kilobase of transcript per Million mapped reads; ^3^ C, control (well-watered conditions); ^4^ 1d, drought treatment for 1 day; ^5^ 2d, drought treatment for 2 days; ^6^ 3d, drought treatment for 3 days; ^7^ log2 ratio, log2(drought treatment/control); ^8^ Species, red letter, up-regulation by drought; blue letter, down-regulation by drought; Abbreviations: At, Arabidopsis thaliana; Bd, Brachypodium distachyon; Gm, Glycine max; Hv, Hordeum vulgare; Mt, Medicago truncatula; Me, Manihot esculenta; Os, Oryza sativa; Pv, Phaseolus vulgaris; Peu, Populus euphratica; Ptc, Populus trichocarpa; Ppe, Prunus persica; Pto, Populus tomentosa; Td, Triticum dicoccoides; Tt, Triticum turgidum; Zm, Zea mays [10, 15]. (XLSX 28 kb) Additional file 8: Tabular data 2. List of drought responsive miRNAs and their putative target genes and their expression patterns of both under drought conditions. (XLSX 101 kb) Additional file 9: Table S4. Drought responsive long noncoding RNAs with their neighboring genes and their expression patterns. (XLSX 67 kb) Additional file 10: Tabular data 3. List of drought responsive long noncoding RNAs (lncRNAs). (XLSX 529 kb) Additional file 11: Figure S4. Drought response phenotype of rice in the vegetative state. **a** The phenotypic effect of progressive drought on wild type rice (*Oryza sativa* cv. Ilmi) at the vegetative growth stage. **b** Decrease in soil water content during drought treatment. **c** The transcript levels of *Dip1* and *RbcS1* in the leaves of drought-treated and well-watered control plants over a time course of exposure to drought were measured by qRT-PCR analysis. Values shown are the means ± SD of three independent experiments and are presented relative to the results from the control. (TIF 17024 kb) Additional file 12: Table S5. Primers used for real time-PCR and the stem-loop RT-PCR miRNA assay. (XLSX 15 kb)

## References

[1] Kang Y, Khan S, Ma X (2009). Climate change impacts on crop yield, crop water productivity and food security. Prog Nat Sci.

[2] Lobell DB, Gourdji SM (2012). The influence of climate change on global crop productivity. Plant Physiol.

[3] Micheletto S, Rodriguez-Uribe L, Hernandez R, Richins RD, Curry V, O’Connell MA (2007). Comparative transcript profiling in roots of Phaseolus acutifolius and P. vulgaris under water deficit stress. Plant Sci.

[4] Todaka D, Nakashima K, Shinozaki K, Yamaguchi-Shinozaki K (2012). Toward understanding transcriptional regulatory networks in abiotic stress responses and tolerance in rice. Rice.

[5] Lee HY, Jang G, Um T, Kim JK, Lee JS, Choi YD (2015). The soluble ABA receptor PYL8 regulates drought resistance by controlling ABA signaling in Arabidopsis. Plant Biotechnol Rep.

[6] Park SH, Jeong JS, Lee KH, Kim YS, Choi YD, Kim JK (2015). OsbZIP23 and OsbZIP45, members of the rice basic leucine zipper transcription factor family, are involved in drought tolerance. Plant Biotechnol Rep.

[7] Todaka D, Shinozaki K, Yamaguchi-Shinozaki K (2015). Recent advances in the dissection of drought-stress regulatory networks and strategies for development of drought-tolerant transgenic rice plants. Front Plant Sci..

[8] Cheong YH, Chang HS, Gupta R, Wang X, Zhu T, Luan S (2002). Transcriptional profiling revels novel interactions between wounding, pathogen, abiotic stress, and hormonal responses in Arabidopsis. Plant Physiol.

[9] Kim ED, Sung S (2012). Long noncoding RNA: unveiling hidden layer of gene regulatory networks. Trends Plant Sci.

[10] Sunkar R, Li YF, Jagadeeswaran G (2012). Functions of microRNAs in plant stress responses. Trends Plant Sci.

[11] Watanabe KA, Ringler P, Gu L, Shen QJ (2014). RNA-sequencing reveals previously unannotated protein- and microRNA-coding genes expressed in aleurone cells of rice seeds. Genomics.

[12] Yoshida T, Mogami J, Yamaguchi-Shinozaki K (2014). ABA-dependent and ABA-independent signaling in response to osmotic stress in plants. Curr Opin Plant Biol..

[13] Khraiwesh B, Zhu JK, Zhu J (2012). Role of miRNAs and siRNAs in biotic and abiotic stress responses of plants. Biochim Biophys Acta.

[14] Kozomara A, Griffiths-Jones S (2014). miRBase: annotating high confidence microRNAs using deep sequencing data. Nucleic Acids Res.

[15] Ferdous J, Hussain SS, Shi BJ (2015). Role of microRNAs in plant drought tolerance. Plant Biotechnol J.

[16] Zhou L, Liu Y, Liu Z, Kong Z, Duan M, Luo L (2010). Genome-wide identification and analysis of drought-responsive microRNAs in Oryza sativa. J Exp Bot.

[17] Ding Y, Tao Y, Zhu C (2013). Emerging roles of microRNAs in the mediation of drought stress response in plants. J Exp Bot.

[18] Li YF, Zheng Y, Addo-Quaye C, Zhang L, Saini A, Jagadeeswaran G, Axtell MJ, Zhang W, Sunkar R (2010). Transcriptome-wide identification of mircoRNA targets in rice. Plant J.

[19] Li WX, Oono Y, Zhu J, He XJ, Wu JM, Iida K, Lu XY, Cui X, Jin H, Zhu JK (2008). The Arabidopsis NFYA5 transcription factor is regulated transcriptionally and posttranscriptionally to promote drought resistance. Plant Cell.

[20] Jeong DH, Green PJ (2013). The role of rice microRNAs in abiotic stress responses. J Plant Biol.

[21] Jeong DH, Park S, Zhai J, Gurazada SG, De Paoli E, Meyers BC, Green PJ (2011). Massive analysis of rice small RNAs: mechanistic implication of regulated microRNAs and variants for differential target RNA cleavage. Plant Cell.

[22] Ponting CP, Oliver PL, Reik W (2009). Evolution and functions of long nonoding RNAs. Cell.

[23] Liu J, Jung C, Xu J, Wang H, Deng S, Bernad L, Arenas-Huertero C, Chua NH (2012). Genome-wide analysis uncovers regulation of long intergenic noncoding RNAs in Arabidopsis. Plant Cell.

[24] Xin M, Wang Y, Yao Y, Song N, Hu Z, Qin D, Xie C, Peng H, Ni Z, Sun Q (2011). Identification and characterization of wheat long non-protein coding RNAs responsive to powdery mildew infection and heat stress by using microarray analysis and SBS sequencing. BMC Plant Biol..

[25] Zhang W, Han Z, Guo Q, Lin Y, Zheng Y, Wu F, Jin W (2014). Identification of maize long non-coding RNAs responsive to drought stress. PLoS One.

[26] German MA, Luo S, Schroth G, Meyers BC, Green PJ (2009). Construction of parallel analysis of RNA ends (PARE) libraries for the study of cleaved miRNA targets and the RNA degradome. Nature Protoc.

[27] Park SH, Chung PJ, Juntawong P, Bailey-Serres J, Kim YS, Jung H, Bang SW, Kim YK, Choi YD, Kim JK (2012). Posttranscriptional control of photosynthetic mRNA decay under stress conditions requires 3′ and 5′ untranslated regions and correlates with differential polysome association in rice. Plant Physiol.

[28] Chung PJ, Kim YS, Park SH, Nahm BH, Kim JK (2009). Subcellular localization of rice histone deacetylases in organelles. FEBS Lett.

[29] Hou CY, Wu MT, Lu SH, Hsing YI, Chen HM (2014). Beyond cleaved small RNA targets: unraveling the complexity of plant RNA degradome data. BMC Genomics..

[30] Cai S, Jiang G, Ye N, Chu Z, Xu X, Zhang J, Zhu G (2015). A key ABA catabolic gene, OsABA8ox3, is involved in drought stress resistance in rice. PLoS One.

[31] Campo S, Baldrich P, Messeguer J, Lalanne E, Coca M, San Segundo B (2014). Overexpression of a calcium-dependent protein kinase confers salt and drought tolerance in rice by preventing membrane lipid peroxidation. Plant Physiol.

[32] Du P, Wu J, Zhang J, Zhao S, Zheng H, Gao G, Wei L, Li Y (2011). Viral infection induces expression of novel phased microRNAs from conserved cellular microRNA precursors. PLoS Pathog.

[33] Qin J, Ma X, Tang Z, Meng Y (2015). Construction of regulatory networks mediated by small RNAs responsive to abiotic stresses in rice (Oryza sativa). Comput Biol Chem..

[34] Zhao B, Liang R, Ge L, Li W, Xiao H, Lin H, Ruan K, Jin Y (2007). Identification of drought-induced microRNAs in rice. Biochem Biophys Res Commun.

[35] Thomson DW, Bracken CP, Goodall GJ (2011). Experimental strategies for microRNA target identification. Nucleic Acids Res.

[36] Liu HH, Tian X, Li YJ, Wu CA, Zheng CC (2008). Microarray-based analysis of stress-regulated microRNAs in Arabidopsis thaliana. RNA.

[37] Wang H, Chung PJ, Liu J, Jang IC, Kean MJ, Xu J, Chua NH (2014). Genome-wide identification of long noncoding natural antisense transcripts and their responses light in Arabidopsis. Genome Res.

[38] Li L, Eichten SR, Shimizu R, Petsch K, Yeh CT, Wu W, Chettoor AM, Givan SA, Cole RA, Fowler JE, Evans MM, Scanlon MJ, Yu J, Schnable PS, Timmermans MC, Springer NM, Muehlbauer GJ (2014). Genome-wide discovery and characterization of maize long noncoding RNAs. Genome Biol.

[39] Zhang YC, Liao JY, Li ZY, Yu Y, Zhang JP, Li QF, Qu LH, Shu WS, Chen YQ (2014). Genome-wide screening and functional analysis identify a large number of long noncoding RNAs involved in the sexual reproduction of rice. Genome Biol.

[40] Zhong S, Joung JG, Zheng Y, Chen YR, Liu B, Shao Y, Xiang JZ, Fei Z, Giovannoni JJ (2011). High-throughput illumine strand-specific RNA sequencing Library preparation. Cold Spring Harb Protoc..

[41] Xu H, Luo X, Qian J, Pang X, Song J, Qian G, Chen J, Chen S (2012). FastUniq: A fast de novo duplicates removal tool for paired short reads. PLoS One.

[42] Varkonyi-Gasic E, Wu R, Wood M, Walton EF, Hellens RP (2007). Protocol: a highly sensitive RT-PCR method for detection and quantification of microRNAs. Plant Methods..

[43] Chen C, Ridzon DA, Broomer AJ, Zhou Z, Lee DH, Nguyen JT, Barbisin M, Xu NL, Mahuvakar VR, Adersen MR, Lao KQ, Livak KJ, Guegler KJ (2005). Real-time quantification of microRNAs by stem-loop RT-PCR. Nucleic Acids Res.

