# Supplementary figures and images for: Transcriptome profiling of drought responsive noncoding RNAs and their target genes in rice
Source: BMC Genomics. 2016 Aug 8;17:563. doi: 10.1186/s12864-016-2997-3 (PMC4977689; doi:10.1186/s12864-016-2997-3)

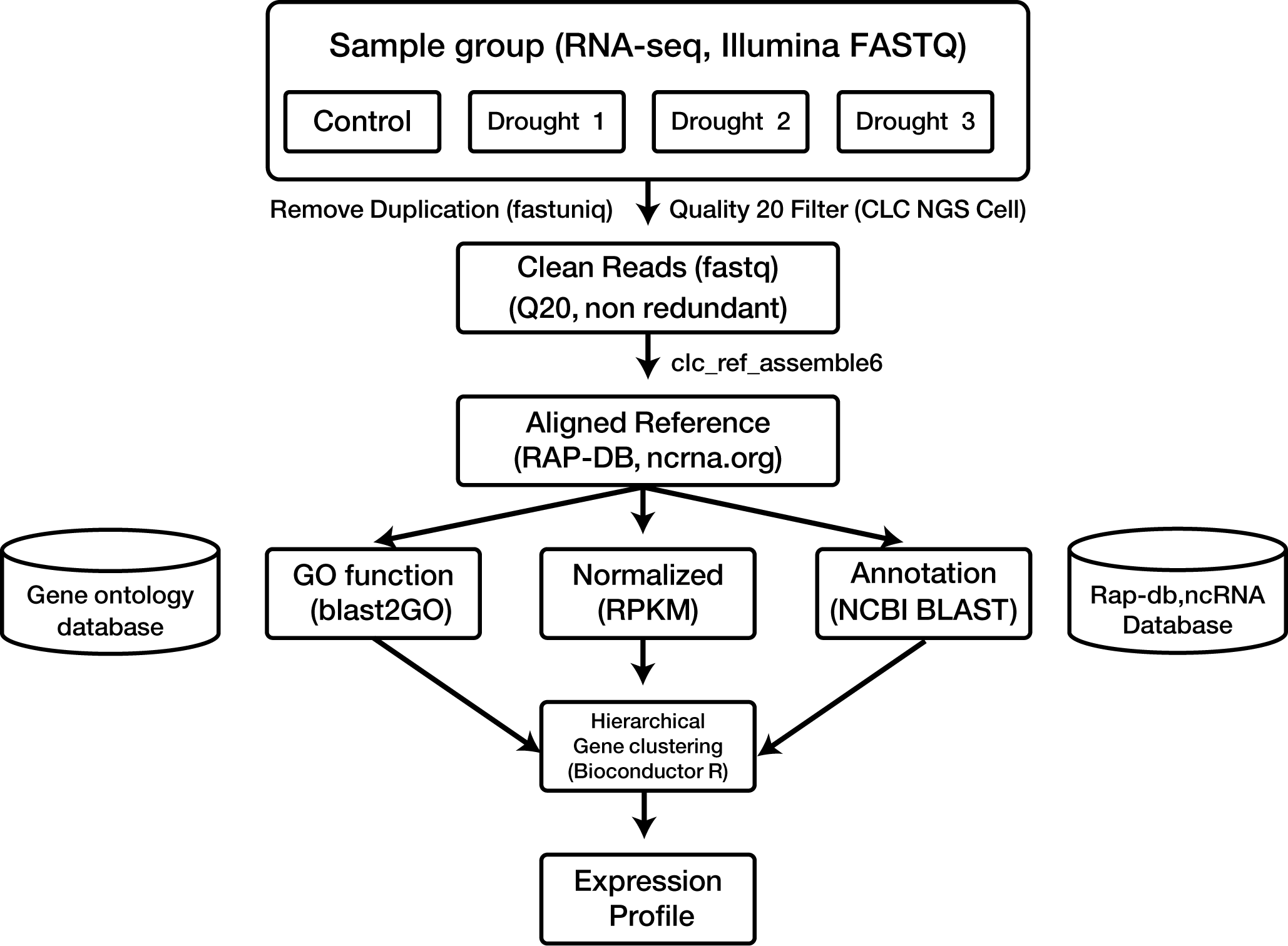

Supplement: Additional file 2: Figure S1. — Flowchart of RNA-seq analysis. (TIF 9814 kb) [file 12864_2016_2997_MOESM2_ESM.tif]

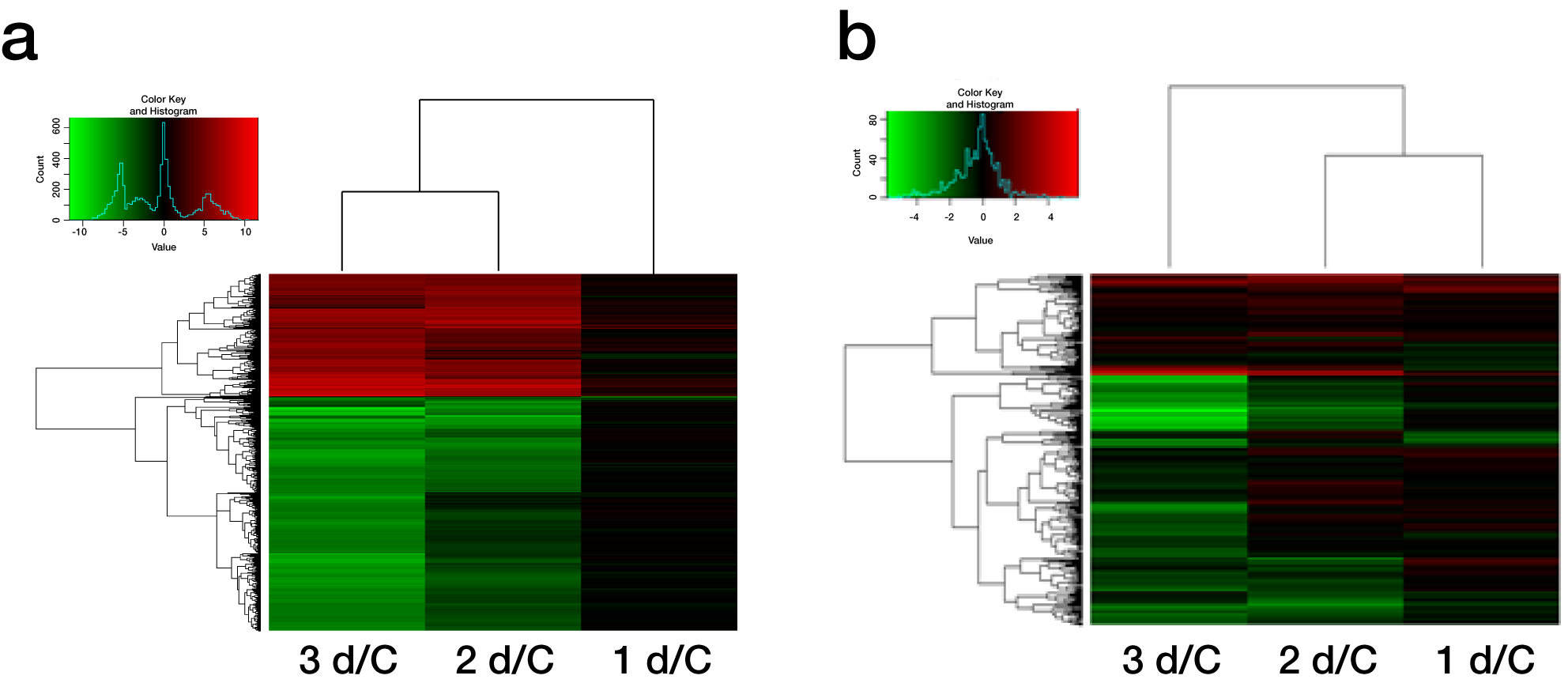

Supplement: Additional file 3: Figure S2. — Heat Map of the differentially expressed coding (a) and noncoding genes (b) under drought conditions. (TIF 5474 kb) [file 12864_2016_2997_MOESM3_ESM.tif]

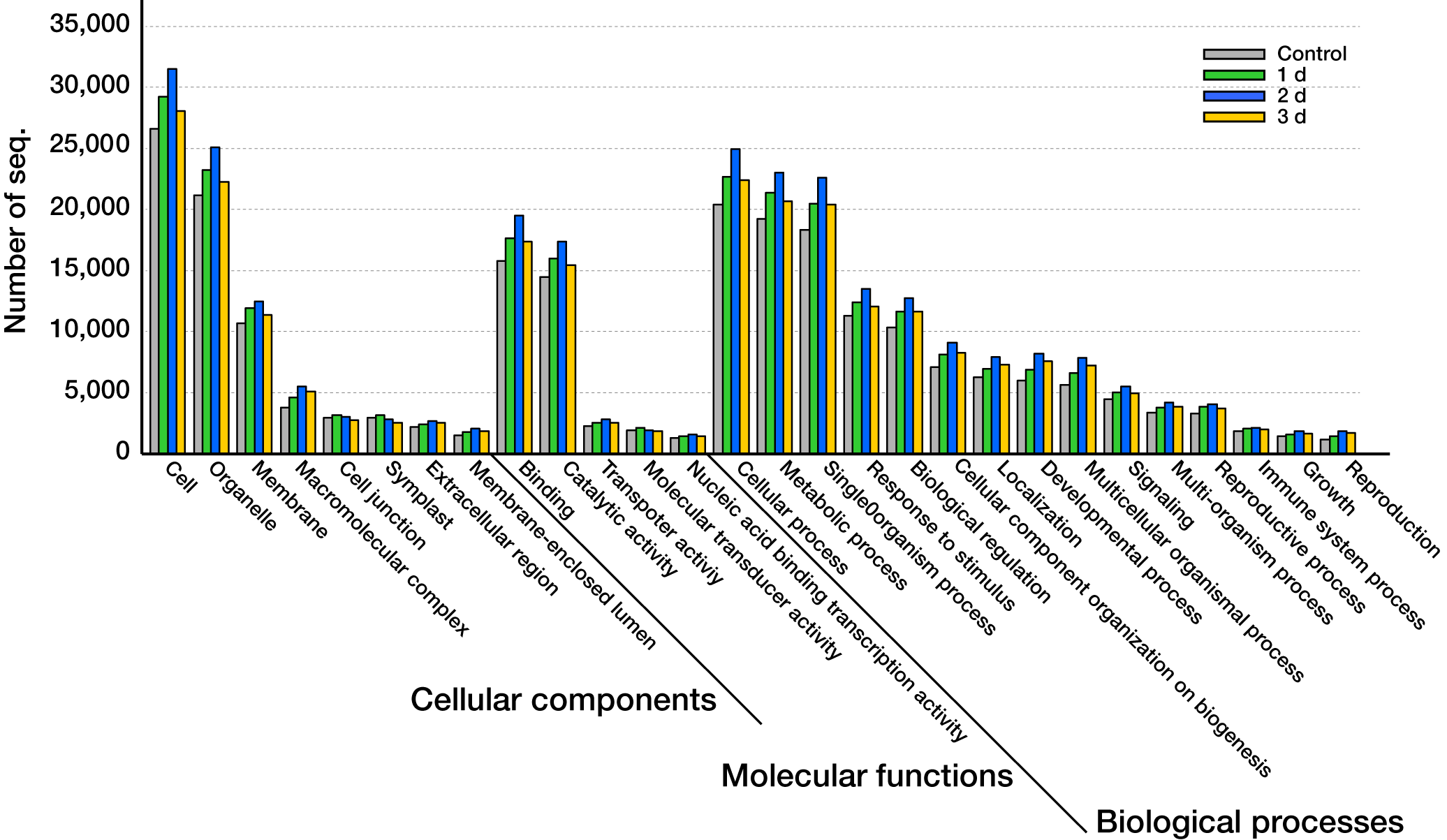

Supplement: Additional file 5: Figure S3. — Differentially expressed transcripts were classified into 3 main GO categories: Biological processes, Cellular components and Molecular functions. (TIF 8355 kb) [file 12864_2016_2997_MOESM5_ESM.tif]

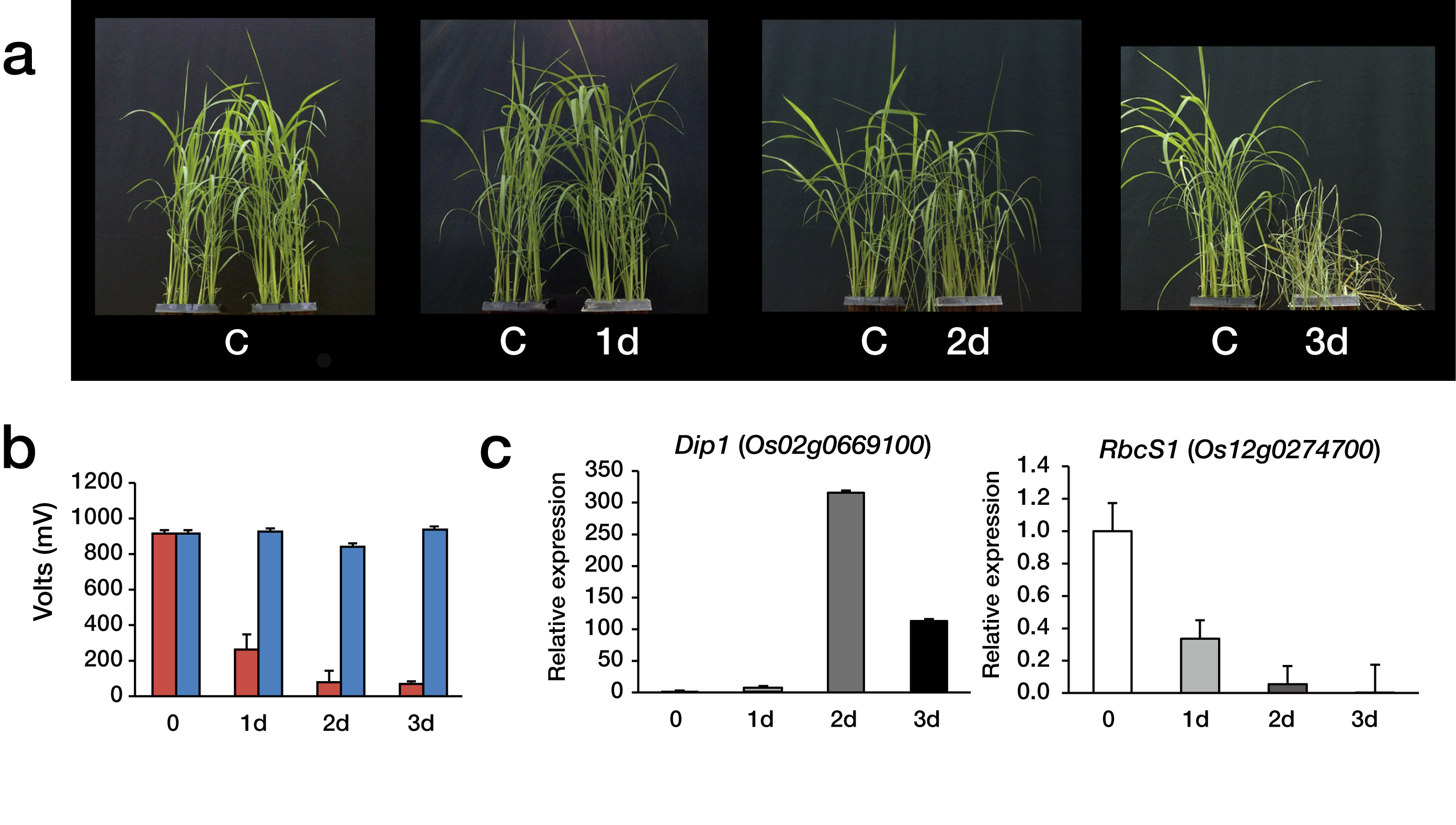

Supplement: Additional file 11: Figure S4. — Drought response phenotype of rice in the vegetative state. a The phenotypic effect of progressive drought on wild type rice (Oryza sativa cv. Ilmi) at the vegetative growth stage. b Decrease in soil water content during drought treatment. c The transcript levels of Dip1 and RbcS1 in the leaves of drought-treated and well-watered control plants over a time course of exposure to drought were measured by qRT-PCR analysis. Values shown are the means ± SD of three independent experiments and are presented relative to the results from the control. (TIF 17024 kb) [file 12864_2016_2997_MOESM11_ESM.tif]
